# From the Cochrane Library: Interventions for Cellulitis and Erysipelas

**DOI:** 10.2196/37888

**Published:** 2022-07-29

**Authors:** Ani Oganesyan, Torunn Sivesind, Robert Dellavalle

**Affiliations:** 1 University of Colorado School of Medicine Aurora, CO United States; 2 Dermatology Service Rocky Mountain Regional Veterans Affairs Medical Center Aurora, CO United States

**Keywords:** erysipelas, cellulitis, cephalosporins, penicillin, macrolides, intervention, dermatology, skin and soft tissue infections, infection, skin, antibiotics, soft tissue, treatment, therapy, bacterial infection

Cellulitis and erysipelas are types of skin and soft tissue infections (SSTIs) that occur when bacteria, commonly group A beta-hemolytic *Streptococcus* and *Staphylococcus*, enter through breaks in the skin. Cellulitis infects the dermis and subcutaneous fat, while erysipelas is a more superficial variant, affecting the superficial dermal lymphatics and adjacent tissues. Untreated, these conditions may result in life-threatening conditions including sepsis, gangrene, or necrotizing fasciitis [1]. Due to the potential risks associated with these conditions, evidence-based research to inform up-to-date treatment guidelines is critical; Table 1 provides guidelines for reference.

A 2010 Cochrane Review [1], “Interventions for Cellulitis and Erysipelas,” assessed 25 randomized controlled trials comparing treatments for primary skin infections, involving a total of 2488 participants. Specifically, the included trials each compared two or more interventions (eg, antibiotics, such as penicillin, macrolides/streptogramins, or cephalosporins, and steroids), routes of administration, and therapy durations. The objective of the review was to assess the efficacy of interventions for nonsurgically acquired SSTIs. This letter will address the limitations of the original review and provide updates based on recent studies.

Macrolides and streptogramins proved superior to penicillin antibiotics in eliminating or reducing cellulitis symptoms (N=2488). Trials comparing oral macrolides against intravenous penicillin found the former to be superior (n=419). No significant differences were found in studies comparing penicillin to cephalosporins (n=88) or among cephalosporin generations (n=538). These comparisons are summarized in Table 2.

Notably, the review [1] highlights a lack of evidence regarding the incorporation of corticosteroids into the antibiotic therapy regimen, whereas subsequent studies have suggested a benefit. The Infectious Disease Society of America states that systemic corticosteroids should be considered in nondiabetic adults to hasten the clinical improvement of cellulitis [2]. A 2018 study [3] assessing corticosteroids (0.5 mg/kg prednisone for 2-3 days) as an add-on therapy to antibiotics for patients hospitalized with erysipelas found that adding steroids resulted in quicker recovery rates and return to full function, with less risk of recurrence [4]. A study of 43 children admitted to the hospital for orbital cellulitis reported a 3-day decrease in length of stay for those treated with adjunctive intravenous dexamethasone (0.3 mg/kg/d every 6 hours for 3 days) compared to those treated with antibiotic monotherapy [3].

Notably, the review [1] did not examine the effectiveness of prophylaxis for cellulitis recurrence; the annual recurrence rate is approximately 8% to 20%. In patients with frequent cellulitis recurrence (3-4 episodes annually), erythromycin, intramuscular penicillin, and oral penicillin VK have been posited as appropriate prophylactic options. A 2021 meta-analysis assessing the use of erythromycin and penicillin found a 69% decreased risk of recurrent cellulitis versus placebo and improved recurrence interval. Penicillin was preferred over erythromycin due to its superior tolerability and cost [5].

**Table 1:** Current Infectious Diseases Society of America guidelines for the management of nonpurulent cellulitis and erysipelas.^a^

Disease entity and antibiotic		Dosage, adults	Dosage, children	Comment
**MSSA^b^ SSTI^c^**				
	Nafcillin or oxacillin	1-2 g every 4 h IV^d^	100-150 mg/kg/d in 4 divided doses	Inactive against MRSA^e^
	Cefazolin	1 g every 8 h IV	50 mg/kg/d in 3 divided doses	For penicillin-allergic patients, except those with immediate hypersensitivity reactions; more convenient than nafcillin with less bone marrow suppression
	Clindamycin	600 mg every 8 h IV or 300-450 mg 4 times daily by mouth	25-40 mg/kg/d in 3 divided doses IV or 25-30 mg/kg/d in 3 divided doses by mouth	Bacteriostatic; potential of cross-resistance and emergence of resistance in erythromycin-resistant strains; inducible resistance in MRSA
	Dicloxacillin	500 mg 4 times daily by mouth	25–50 mg/kg/d in 4 divided doses by mouth	Oral agent of choice for methicillin-susceptible strains in adults; rarely used in pediatrics
	Cephalexin	500 mg 4 times daily by mouth	25-50 mg/kg/d 4 divided doses by mouth	For penicillin-allergic patients except those with immediate hypersensitivity reactions; the availability of a suspension and requirement for less frequent dosing
	Doxycycline, minocycline	100 mg twice daily by mouth	Not recommended for age <8 y	Bacteriostatic; limited recent clinical experience
	Trimethoprim-sulfamethoxazole	1-2 double-strength tablets twice daily by mouth	8-12 mg/kg (based on trimethoprim component) in either 4 divided doses IV or 2 divided doses by mouth	Bactericidal; efficacy poorly documented
**MRSA SSTI**				
	Vancomycin	30 mg/kg/d in 2 divided doses IV	40 mg/kg/d in 4 divided doses IV	For penicillin-allergic patients; parenteral drug of choice for treatment of infections caused by MRSA
	Linezolid	600 mg every 12 h IV or 600 mg twice daily by mouth	10 mg/kg every 12 h IV or by mouth for children <12 y	Bacteriostatic; limited clinical experience; no cross-resistance with other antibiotic classes; costly
	Clindamycin	600 mg every 8 h IV or 300-450 mg 4 times daily by mouth	25-40 mg/kg/d in 3 divided doses IV or 30-40 mg/kg/d in 3 divided doses by mouth	Bacteriostatic; potential of cross-resistance and emergence of resistance in erythromycin-resistant strains; inducible resistance in MRSA; important option for pediatrics
	Daptomycin	4 mg/kg every 24 h IV	N/A^f^	Bactericidal; possible myopathy
	Ceftaroline	600 mg twice daily IV	N/A	Bactericidal
	Doxycycline, minocycline	100 mg twice daily by mouth	Not recommended for age <8 y	Bacteriostatic; limited recent clinical experience
	Trimethoprim-sulfamethoxazole	1-2 double-strength tablets twice daily by mouth	8–12 mg/kg/d (based on trimethoprim component) in either 4 divided doses IV or 2 divided doses by mouth	Bactericidal; limited published efficacy data
Streptococcal skin infections		Penicillin: 2-4 million units every 4-6 h IV; Clindamycin: 600-900 mg every 8 h IV; Nafcillin: 1-2 g every 4-6 h IV; Cefazolin: 1 g every 8 h IV; Penicillin: VK 250-500 mg every 6 h by mouth; Cephalexin 500 mg every 6 h by mouth	Penicillin: 60,000-100,000 units/kg/dose every 6 h; 10-13 mg/kg dose every 8 h IV; 50 mg/kg/dose every 6 h; 33 mg/kg/dose every 8 h IV	N/A

^a^Recommendation according to the Infectious Diseases Society of America. Doses listed are not appropriate for neonates. Infection due to *Staphylococcus* and *Streptococcus* species. Duration of therapy is 7 days depending on the clinical response.

^b^MSSA: methicillin-susceptible *Staphylococcus aureus*.

^c^SSTI: skin and soft tissue infection.

^d^IV: intravenous.

^e^MRSA: methicillin-resistant *Staphylococcus aureus*.

^f^N/A: not applicable.

**Table 2:** Treatment comparison with respective results, risk ratio, and CI.^a^

Comparison	Measurement	Results	RR^b^ (95% CI)	Studies, n	Patients, n
Macrolides/streptogramins vs penicillin antibiotics	Symptoms rated by participant or medical practitioner	Macrolides/streptogramins were superior	0.84 (0.73-0.97)	25	2488
Oral macrolide vs IV^c^ penicillin	Symptoms rated by participant or medical practitioner	Oral therapy was superior	0.85 (0.73-0.98)	3	419
Penicillin vs cephalosporin	Symptoms rated by participant or medical practitioner	No difference in treatment effect	0.99 (0.68-1.43)	3	88
Cephalosporin vs cephalosporin^d^	Symptoms rated by participant or medical practitioner	No difference in treatment effect	1.00 (0.94-1.06)	6	538

^a^Primary outcomes included symptoms rated by participant or medical practitioner (eg, duration and intensity of fever, pain, redness of the affected area, swelling of the skin surface and subcutaneous tissue, blister formation), proportion symptom‐free (*cure*), and at a time specified by the study authors), the proportion with severe complications (eg, severe sepsis, multi-organ failure, or death), and quality of life scores (ie, generic and disease-specific items and return to normal activity).

^b^RR: relative risk.

^c^IV: intravenous.

^d^Aggregate data from studies evaluating the following cephalosporins: ceftriaxone, cefdinir, cefonicid, cefditoren, cefadroxil, and cefuroxime.

The review reported insufficient data to determine the ideal duration of therapy. International recommendations for treatment duration in SSTIs are inconsistent (5-14 days) [2]—however, this is largely based on expert opinion, with few randomized controlled trials evaluating this parameter. Future research should address this limitation to maximize patient benefit while reducing the effects of prolonged exposure.

## Abbreviations

SSTI: skin and soft tissue infection
